# Microbial Dynamics in Periodontal Regeneration: Understanding Microbiome Shifts and the Role of Antifouling and Bactericidal Materials: A Narrative Review

**DOI:** 10.3390/cimb46110724

**Published:** 2024-10-30

**Authors:** Nada Tawfig Hashim, Rasha Babiker, Sivan Padma Priya, Riham Mohammed, Nallan CSK Chaitanya, Vivek Padmanabhan, Shadi El Bahra, Muhammed Mustahsen Rahman, Bakri Gobara Gismalla

**Affiliations:** 1 RAK College of Dental Sciences, RAK Medical & Health Sciences University, Ras Al Khaimah P.O. Box 12973, United Arab Emirates; sivan.padma@rakmhsu.ac.ae (S.P.P.); riham.abdelraouf@rakmhsu.ac.ae (R.M.); krishna.chytanya@rakmhsu.ac.ae (N.C.C.); vivekpadmanabhan@rakmhsu.ac.ae (V.P.); shadi.elbahra@rakmhsu.ac.ae (S.E.B.); mustahsen@rakmhsu.ac.ae (M.M.R.); 2 RAK College of Medical Sciences, RAK Medical & Health Sciences University, Ras Al Khaimah P.O. Box 11172, United Arab Emirates; rashababiker@rakmhsu.ac.ae; 3 Faculty of Dentistry, University of Khartoum, Khartoum 11115, Sudan; bakrigobara10@gmail.com

**Keywords:** periodontal regeneration, oral microbiome, bone grafting, guided tissue regeneration, antifouling materials, bactericidal materials, microbial balance, biofilm disruption

## Abstract

Periodontal regeneration is a multifaceted therapeutic approach to restore the tooth-supporting structures lost due to periodontal diseases. This manuscript explores the intricate interactions between regenerative therapies and the oral microbiome, emphasizing the critical role of microbial balance in achieving long-term success. While guided tissue regeneration (GTR), bone grafting, and soft tissue grafting offer promising outcomes in terms of tissue regeneration, these procedures can inadvertently alter the oral microbial ecosystem, potentially leading to dysbiosis or pathogenic recolonization. Different grafting materials, including autografts, allografts, xenografts, and alloplasts, influence microbial shifts, with variations in the healing timeline and microbial stabilization. Biologics and antimicrobials, such as enamel matrix derivatives (EMD) and sub-antimicrobial dose doxycycline (SDD), play a key role in promoting microbial homeostasis by supporting tissue repair and reducing pathogenic bacteria. Emerging strategies, such as enzyme-based therapies and antifouling materials, aim to disrupt biofilm formation and enhance the effectiveness of periodontal treatments. Understanding these microbial dynamics is essential for optimizing regenerative therapies and improving patient outcomes. The future of periodontal therapy lies in the development of advanced materials and strategies that not only restore lost tissues but also stabilize the oral microbiome, ultimately leading to long-term periodontal health.

## 1. Introduction

**Figure 1:**
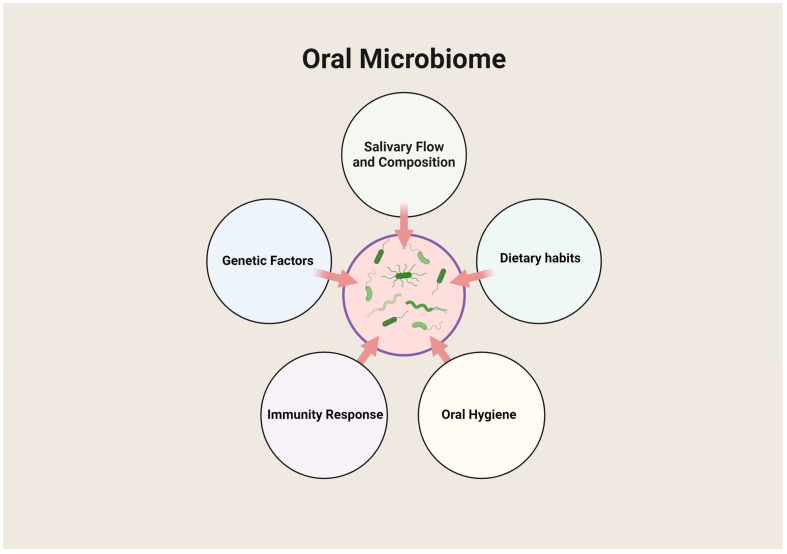
The complex interplay of factors that influence the oral microbiome, a community of microorganisms residing in the mouth. Salivary flow and composition and dietary habits play a significant role in regulating microbial growth, while effective oral hygiene helps control harmful bacteria. The immune response is crucial in maintaining microbial balance, and genetic factors can predispose individuals to specific oral health conditions, affecting the overall composition of the oral microbiome. These interconnected factors collectively determine the health and stability of the oral environment. Created by Biorender.com.

**Figure 2:**
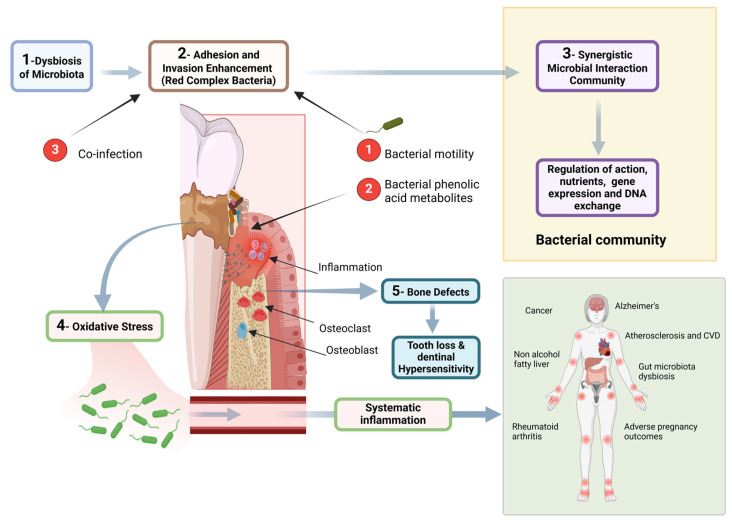
The complex pathological process of periodontal disease highlights the progression from microbial dysbiosis to systemic inflammation. It begins with the dysbiosis of the oral microbiota, where an imbalance in bacterial populations favors the growth of harmful pathogens, such as the red complex bacteria. These pathogens enhance adhesion and invasion into the periodontal tissues, leading to a local inflammatory response. As a result, oxidative stress is generated, further exacerbating tissue damage. The inflammatory process promotes bone defects due to the activity of osteoclasts, leading to tooth loss and dentinal hypersensitivity. Additionally, persistent inflammation can spread systemically, contributing to a range of systemic conditions, including cardiovascular disease, Alzheimer’s, and rheumatoid arthritis, illustrating the far-reaching impacts of periodontal disease beyond the oral cavity. Created by Biorender.com.

The oral microbiome is a dynamic ecosystem consisting of over 700 species of bacteria, alongside fungi, viruses, and protozoa, inhabiting various niches like the tongue, cheeks, palate, teeth, and gingival sulcus. Commensal bacteria in the oral cavity play a crucial role in preventing colonization by pathogens, known as colonization resistance [1]. In a healthy state, this microbiome has a symbiotic relationship with the host, contributing to immunological priming, regulation of inflammation, and protection against harmful microbes [2]. Disruptions in this balance can lead to periodontal diseases such as gingivitis and periodontitis [3]. Factors like salivary flow, diet, oral hygiene, immune responses, and genetics influence the stability of the oral microbiome [4]. Saliva provides nutrients and antimicrobial proteins, while dietary habits, especially high sugar intake, favor the growth of bacteria responsible for dental caries and periodontal disease [5]. Poor oral hygiene leads to plaque accumulation, creating a reservoir for pathogenic bacteria [6]. Secretory IgA and other immune components modulate bacterial populations [7]. Figure 1 demonstrates these interactions within the oral environment. The transition from a healthy microbiome to a diseased state involves complex interactions, including dysbiosis—a shift from a symbiotic to a pathogenic microbial community, marked by species such as *Porphyromonas gingivalis*, *Tannerella forsythia*, and *Treponema denticola* [8]. These bacteria form biofilms resistant to host defenses and antimicrobial treatments, leading to chronic inflammation [9]. Pathogenic bacteria stimulate inflammatory responses via Toll-like receptors (TLRs), resulting in tissue destruction, including the degradation of extracellular matrix components by matrix metalloproteinases (MMPs) [10,11]. Figure 2 illustrates the microbial interactions and the biofilm formation in periodontal disease.

**Figure 3:**
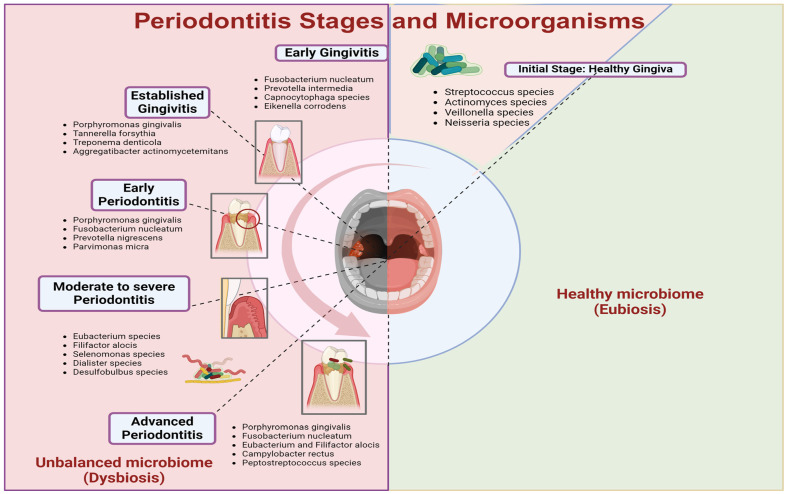
The progression of periodontitis from healthy gingiva to advanced stages of the disease, highlighting the associated changes in the oral microbiome. Initially, in a healthy gingival state, the microbiome is balanced (eubiosis), consisting mainly of beneficial bacteria such as *Streptococcus*, *Actinomyces*, *Veillonella*, and *Neisseria* species. As the disease progresses to early gingivitis, there is an increase in pathogenic bacteria like *Fusobacterium nucleatum* and *Prevotella intermedia*. Established gingivitis sees the further dominance of pathogens such as *Porphyromonas gingivalis* and *Treponema denticola*. These harmful bacteria proliferate in early periodontitis, leading to deeper periodontal pockets and more significant tissue damage. As the condition advances to moderate to severe periodontitis, more aggressive bacteria like *Eubacterium* and *Filifactor alocis* emerge, exacerbating tissue destruction and bone loss. Finally, in advanced periodontitis, a highly unbalanced microbiome (dysbiosis) dominates, with pathogens like *Campylobacter rectus* and *Peptostreptococcus* species, leading to severe periodontal destruction and potential tooth loss. Created by Biorender.com.

**Figure 4:**
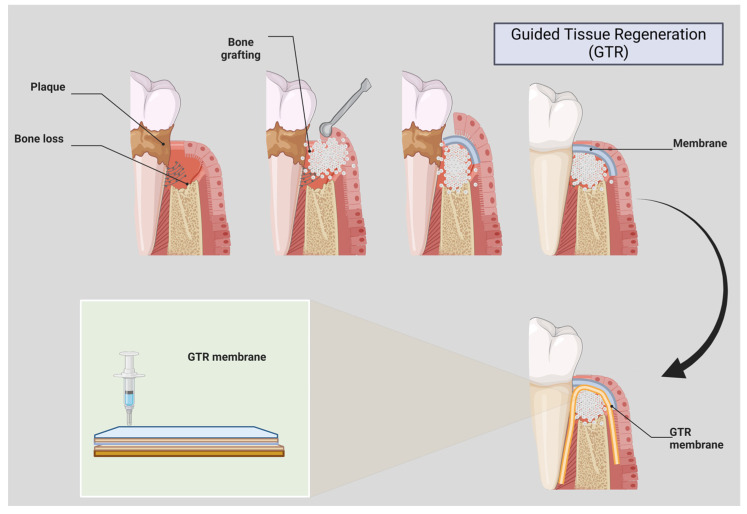
The process of Guided Tissue Regeneration (GTR), a periodontal therapy used to restore lost bone and supporting tissues around teeth affected by periodontal disease. Initially, the figure shows a tooth with significant bone loss due to the destructive effects of plaque accumulation. The next step involves the placement of bone graft material into the defect, which acts as a scaffold to support new bone formation. Following this, a GTR membrane is carefully placed over the grafted area to prevent the invasion of soft tissue, ensuring that only bone and periodontal tissues regenerate in the space. Over time, this approach promotes the regeneration of lost bone and periodontal structures, ultimately restoring the tooth’s stability and improving overall oral health. Created by Biorender.com.

Periodontitis, a chronic inflammatory disease, damages the tooth-supporting structures such as the periodontal ligament, cementum, and alveolar bone. Research shows that shifts in the oral microbiome, particularly the rise of periodontitis-associated species like *P. gingivalis*, have been influenced by changes in diet since the Neolithic period [12]. Unlike infections caused by a specific pathogen, periodontitis results from complex interactions between microbial communities and host factors, marked by an imbalance in immune responses, including inappropriate shifts between Th17 and T regulatory cells [13]. This dysregulation drives chronic inflammation and tissue destruction, as shown in Figure 3. Recent advancements in microbiome analysis, including next-generation sequencing (NGS), have provided greater accuracy in characterizing subgingival microbial communities during different stages of periodontal health and disease [12]. In health, the subgingival microbiome is dominated by commensal species like *Streptococcus sanguinis*, *Actinomyces*, and *Veillonella*, which help maintain a stable biofilm and regulate acid production [12]. As gingivitis progresses, Gram-negative anaerobes such as *Fusobacterium nucleatum* and *Prevotella intermedia* increase, facilitating the adhesion of more pathogenic species [12]. Table 1 summarizes these microbial shifts across different stages of periodontal disease. As periodontitis advances, pathogenic species like *P. gingivalis*, *T. forsythia*, and *T. denticola* dominate the subgingival microbiome, destroying tissues [14]. In severe stages, microbial diversity shifts further, with species like *Filifactor alocis* and *Campylobacter rectus* contributing to alveolar bone resorption and advanced periodontal damage [13,14,15]. Figure 4 demonstrates these microbial shifts and their correlation with disease progression.

**Figure 5:**
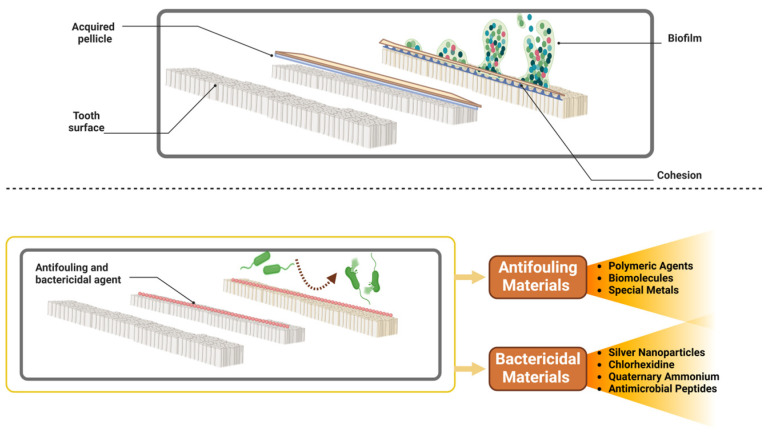
The formation and prevention of dental biofilm on tooth surfaces. The upper section of the figure shows the typical progression of biofilm development, starting with the formation of an acquired pellicle on the tooth surface. This pellicle allows initial bacterial adhesion, followed by the proliferation of bacteria and the maturation of the biofilm, leading to a cohesive microbial community that is challenging to remove. The lower section demonstrates antifouling and bactericidal materials to prevent biofilm formation. Antifouling materials, such as polymeric agents, biomolecules, and special metals inhibit bacterial adhesion to the tooth surface. Bactericidal materials, including silver nanoparticles, chlorhexidine, quaternary ammonium compounds, and antimicrobial peptides actively kill bacteria that come into contact with the treated surface, thereby preventing biofilm formation and maintaining oral health. Created by Biorender.com.

The treatment of periodontitis requires addressing the destruction of periodontal tissues and correcting the microbial imbalance. Traditional therapies like scaling and root planing aim to remove biofilms, but regenerative treatments, including guided tissue regeneration (GTR), bone grafting, and biologics like enamel matrix derivatives, focus on restoring both tissue and microbial homeostasis [12,13]. Figure 5 illustrates the approach of using regenerative therapies to rebalance the oral microbiome and promote healing.

Addressing microbial dynamics in periodontal regeneration is crucial, as the microbiome plays a significant role in modulating tissue healing and immune responses. Advanced therapeutic strategies aimed at disrupting pathogenic biofilms while promoting the restoration of a healthy, symbiotic microbiome hold great potential for improving treatment outcomes and ensuring long-term periodontal health [12,13].

The present review focuses on the emerging relationship between the oral microbiome and periodontal tissue regeneration. This is a relatively new area of research that investigates how microbial taxa associated with the oral cavity might influence or modulate the regeneration of periodontal tissues, including the gingiva, periodontal ligament, cementum, and alveolar bone. The review aims to explore how these microbial dynamics interact with host immune responses and affect the success of regenerative therapies such as guided tissue regeneration (GTR), bone grafting, and the use of biologics. To conduct this review, we systematically searched the PubMed database (https://pubmed.ncbi.nlm.nih.gov/, accessed on 20 October 2024) using keywords including “microbiota”, “microbiome”, “periodontal regeneration”, “oral dysbiosis”, and “bone grafting”. We focused on articles that provided insights into the interactions between the microbiota and periodontal healing processes. Additionally, the inclusion criteria were articles that discussed microbial shifts in both health and disease states, their impact on tissue regeneration, and the role of antifouling and bactericidal materials in regenerative treatments. We highlighted animal studies and human clinical trials that provided relevant data to inform our analysis. In particular, we have emphasized research on the microbiome–host associations in periodontal tissue healing and how these relationships may shape the outcomes of regenerative therapies. This review serves to present the current understanding of microbial dynamics in periodontal regeneration and explores potential therapeutic strategies to harness the microbiome for enhancing tissue healing and long-term periodontal health.

## 2. Microbial Dynamics in Periodontal Regeneration

Over the last few decades, the primary goal of periodontal therapy has shifted from repair to reconstruction of these damaged tissues to reverse the effects of the disease process [16]. One of the key approaches in this pursuit of “true periodontal regeneration” has been using various bone graft materials, which have shown significant clinical improvements in probing depth reduction, attachment gain, and bone regeneration [17]. Moreover, the introduction of various biomaterials and grafting techniques in the oral cavity can have a significant impact on the oral microbiome, which plays a crucial role in the initiation and progression of periodontal diseases [18,19]. The alteration of the delicate balance within the oral microbial community can lead to a shift towards a more pathogenic profile, potentially undermining the long-term success of regenerative procedures [20]. Regenerative periodontal therapy has been a topic of intense research and clinical interest for the past three decades. While significant progress has been made, the unpredictability of the regenerative process and the poor understanding of the origin and nature of the new tissues formed have made it challenging to design effective and reliable clinical trials [21]. One key area that warrants further investigation is the impact of bone and tissue regeneration procedures on the oral microbiome. Understanding how these interventions affect the delicate balance of the oral microbial ecosystem can provide valuable insights into the long-term outcomes of regenerative therapies and guide the development of more holistic treatment approaches that address both the structural and microbiological aspects of periodontal diseases [22]. Guided Tissue Regeneration (GTR) is a surgical procedure that promotes the growth of new periodontal tissues. It involves placing a barrier membrane between the gum and the bone to direct the growth of new bone and soft tissue [23]. The membrane prevents the fast-growing epithelial cells from occupying the space needed for bone and ligament growth. The surgical area is cleaned, and the barrier membrane is placed over the defect. The membrane can be resorbable or non-resorbable, depending on the clinical situation. The flap is then sutured back into place. GTR promotes selective cell repopulation, allowing for the regeneration of periodontal ligament, cementum, and alveolar bone. However, the technique requires precise surgical skills and post-operative care, and the success of GTR is also dependent on patient factors, such as oral hygiene and smoking status [24,25] Figure 4. 

Bone grafting is a common procedure used to treat bony defects in periodontal disease. It involves the placement of bone graft material into the defect to promote bone regeneration. Types of bone grafts include autografts (bone taken from the patient’s own body, usually from the hip or another area of the mouth), allografts (bone taken from a donor), xenografts (bone taken from another species, usually bovine), and alloplasts (synthetic bone graft materials) [26]. The surgical site is prepared, and the bone graft material is placed into the defect. The area is then covered with a barrier membrane, if necessary, and the flap is sutured back into place. Bone grafting can significantly enhance the regeneration of bone and improve the stability of teeth. However, the procedure can be invasive, and there is a risk of infection and graft rejection [27].

Soft tissue grafting is used to treat gingival recession and involves the placement of soft tissue grafts to cover exposed roots and increase the amount of keratinized tissue. Types of soft tissue grafts include connective tissue grafts (tissue taken from under the palatal mucosa), free gingival grafts (tissue taken from the surface of the palate), and pedicle grafts (tissue that is partially detached and moved to cover the recession defect) [28]. The graft tissue is harvested and placed over the area of recession. The graft is then secured with sutures. Soft tissue grafting can improve the aesthetics of the smile, reduce sensitivity, and increase the amount of keratinized tissue. However, the procedure requires careful surgical technique and post-operative care to ensure the success of the graft [29].

## 3. Exploring the Impact of Regenerative Therapies on the Oral Microbial Ecosystem

The impact of regenerative therapies on the oral microbial ecosystem is a critical yet often overlooked aspect of periodontal treatment [22]. Regenerative periodontal therapies, such as guided tissue regeneration (GTR), bone grafting, and the use of biologics, are primarily aimed at restoring the tooth-supporting structures lost to periodontal disease [30]. However, these interventions can also influence the oral microbiome, which is fundamental in maintaining periodontal health [31]. While regenerative therapies focus on rebuilding tissue, they inadvertently alter the local environment, reducing inflammation and improving oxygenation, which can shift the microbial balance back toward health-associated species such as *Streptococcus sanguinis* and *Actinomyces naeslundii* [32]. This microbial shift is crucial for the long-term success of regenerative procedures, as a stable, health-associated microbiome is essential for preventing reinfection and promoting sustained tissue regeneration [32]. Thus, understanding and managing the changes in the oral microbiome following regenerative treatments is vital for achieving not only structural repair but also lasting microbial and periodontal health.

## 4. General Microbiome Changes After GTR

Before GTR, pathogenic species like *Porphyromonas gingivalis*, *Tannerella forsythia*, and *Treponema denticola* dominate due to their ability to thrive in anaerobic, inflamed environments typical of periodontitis [33]. These bacteria are adept at evading immune responses and contributing to tissue destruction, which supports their growth in periodontal pockets [34]. After guided tissue regeneration (GTR), the local environment experiences a substantial shift due to the reduction of periodontal pocket depth, increased oxygen levels, and decreased inflammation [35]. This transition fosters a more aerobic environment, which supports the growth of beneficial species like *Streptococcus sanguinis* and *Actinomyces naeslundii*. The mechanism behind this shift lies in the ability of these bacteria to thrive in oxygenated environments and establish biofilms that resist pathogenic recolonization [36]. *Streptococcus sanguinis* is an early colonizer that plays a crucial role in maintaining oral health by producing extracellular polysaccharides that help stabilize the biofilm matrix. This bacterium promotes a low-inflammatory state by producing hydrogen peroxide (H_2_O_2_), which inhibits the growth of anaerobic pathogens like *Porphyromonas gingivalis* and *Tannerella forsythia* [37]. Additionally, *S. sanguinis* competes for nutrients and adhesion sites, preventing the recolonization of harmful bacteria. Its production of bacteriocins—antimicrobial peptides—directly inhibits the growth of nearby pathogens, thus reinforcing the dominance of beneficial bacteria in the healing biofilm [38]. Similarly, *Actinomyces naeslundii* plays a pivotal role in maintaining biofilm health and stabilizing the microbial community. This Gram-positive facultative anaerobe is particularly adept at metabolizing complex carbohydrates and producing lactic acid, which lowers the pH and prevents the overgrowth of pathogenic species [39]. 

*A. naeslundii* also facilitates the adhesion of other commensal bacteria to the tooth surface through co-aggregation mechanisms, which enhance biofilm cohesion and resilience against environmental perturbations, such as shifts in nutrient availability or changes in local pH [40]. *Veillonella parvula* reappears in greater numbers due to its ability to metabolize lactic acid produced by other bacteria, further reducing acidity and promoting a healthy biofilm that supports tissue regeneration [41].

## 5. General Microbiome Changes After Bone Grafting

The oral microbiome plays a crucial role in the healing process following periodontal regeneration with different bone grafts, such as allografts, autografts, xenografts, and alloplasts [42]. While each bone graft type influences the local microbiome differently, there are some similarities in their effects on bacterial composition, especially when considering factors like the healing environment, oxygenation, and inflammation levels. However, there are also distinct impacts related to specific graft properties [43]. Across all graft types, a successful periodontal regeneration procedure typically reduces pocket depth, increases tissue attachment, and stabilizes the local oral environment [36]. These improvements create a more oxygenated and less inflammatory environment, which supports the growth of health-associated bacteria and discourages the proliferation of anaerobic periodontal pathogens [36]. After regeneration procedures, there is typically a shift from a dysbiotic microbiome dominated by pathogens like *Porphyromonas gingivalis*, *Tannerella forsythia*, and *Treponema denticola* (common in periodontitis) to a more balanced microbiome dominated by beneficial commensal bacteria [36].

Health-associated bacteria that often increase following successful periodontal regeneration include *Streptococcus sanguinis* and *Streptococcus gordonii*, early colonizers promoting biofilm stability and oral health [44]. These bacteria help prevent pathogenic recolonization by producing hydrogen peroxide, which inhibits the growth of anaerobic bacteria. Additionally, *Actinomyces naeslundii* plays a crucial role in maintaining biofilm structure and contributes to a low-inflammatory state in periodontal tissues, further promoting healing [45]. *Veillonella* species also contribute to biofilm health by metabolizing lactic acid produced by other bacterial species, preventing acid buildup and helping to stabilize the biofilm environment. However, the specific bone graft type used can influence how these microbial changes occur and the timeline of microbial shifts, with some grafts promoting faster microbial stabilization than others [41].

## 6. Autografts (Autogenous Bone Grafts)

Autografts are derived from the patient’s tissue, reducing the risk of an immune response or graft rejection. Since autografts provide viable cells (osteogenic potential), they typically promote faster tissue regeneration, which helps reduce inflammation and stabilize the local microbiome more rapidly [46]. The rapid healing environment created by autografts generally favors the recolonization of beneficial commensal bacteria like *Streptococcus sanguinis* and *Actinomyces naeslundii* over pathogenic species. Autografts tend to have the least impact on the oral microbiome compared to other graft types because they integrate more naturally with the host tissue [26].

The faster healing with autografts minimizes the window of time during which pathogens like *P. gingivalis* could recolonize, contributing to a more stable microbial environment post-regeneration [26]. The influence of the graft material on the microbiome is also noteworthy. Autologous grafts, derived from the patient’s tissue, tend to promote faster microbial stabilization compared to allografts or synthetic materials [47]. This is because autologous tissue integrates more quickly with the host, reducing the window during which opportunistic pathogens could colonize the graft site. As the new tissue becomes vascularized and integrated, the local immune system helps to maintain microbial balance by preventing the overgrowth of pathogenic species [47]. What is particularly new and intriguing is the role of immunomodulatory factors released during the healing process, which have been shown to further enhance microbial homeostasis. These factors help maintain a balanced immune response, minimizing inflammation and promoting the growth of beneficial commensal bacteria [48]. Future research is exploring whether specific bioactive molecules within the graft material can accelerate the establishment of a health-associated microbiome, potentially leading to improved clinical outcomes and long-term graft stability.

## 7. Allografts (Donor Bone Grafts)

Allografts are processed to be sterile, which ensures they are free of pathogens but also delays their integration compared to autografts. The slower resorption and integration process with allografts can prolong the period of microbial instability [49]. Initially, there may be an increased risk of bacterial colonization on the graft site, especially if the local environment is not fully stabilized. However, over time, as the graft integrates, the microbiome shifts towards a composition similar to that of autografts, with increased levels of *Streptococcus* and *Actinomyces* species [50]. Allografts may require additional attention to infection control in the early postoperative period due to the delayed healing process, which could allow opportunistic pathogens to colonize if oral hygiene is inadequate [50].

## 8. Xenografts (Animal-Derived Bone Grafts)

Xenografts, typically derived from bovine or porcine sources, require extensive processing to prevent immune reactions, which also slows their integration. The slow resorption of xenografts creates a longer period in which the oral microbiome is in flux [51]. While xenografts are highly osteoconductive, their prolonged presence in the oral cavity may encourage biofilm formation, potentially by bacteria like *Fusobacterium nucleatum* or *Prevotella* species. However, with good infection control, the microbiome eventually stabilizes to resemble that of the other graft types, with an increase in beneficial species such as *Streptococcus gordonii* and *Veillonella* [52]. Due to the extended resorption period of xenografts, there is a higher risk of bacterial colonization during the initial healing phase compared to other graft types. Clinicians need to monitor for potential infections or dysbiosis more closely in the early stages of healing [53]. 

## 9. Alloplasts (Synthetic Bone Grafts)

Alloplastic grafts, composed of synthetic materials like hydroxyapatite or bioactive glass, have a neutral impact on the oral microbiome due to their inert nature [43]. These grafts do not induce an immune response and do not carry pathogens. However, their slow resorption and longer presence in the oral cavity could lead to a temporary increase in biofilm formation, particularly from early colonizers like *Streptococcus mutans* or *Actinomyces* species, which might lead to surface-level biofilm on the synthetic material [54].

The bioinert nature of alloplastic grafts means that they are less likely to promote aggressive immune responses or pathogen colonization compared to xenografts, but their long presence could act as a surface for biofilm formation, requiring consistent post-operative monitoring [55].

## 10. General Microbiome Changes After Soft Tissue Grafting

Following soft tissue grafting, the immediate post-surgical period is marked by a shift in the microbial community as the newly placed graft integrates with the host tissue [56]. 

The grafting process itself temporarily disrupts the local microbial balance, creating a vulnerable environment that can be prone to pathogenic recolonization if infection control is inadequate [57]. 

Initially, a decrease in pathogenic bacteria such as *Porphyromonas gingivalis* and *Tannerella forsythia* is observed due to the removal of diseased tissue during surgery. This reduction in anaerobic, pathogenic bacteria paves the way for beneficial changes in the oral microbiome. As healing progresses, health-associated bacteria, such as *Streptococcus sanguinis*, *Streptococcus gordonii*, and *Veillonella* species, begin to recolonize the graft site. These early colonizers are critical for establishing a healthy biofilm that resists pathogenic invasion [58]. 

## 11. The Role of Biologics, Antimicrobials, and Microbiome Modulation in Periodontal Regeneration

Biologics, such as enamel matrix derivatives (EMD), platelet-derived growth factors (PDGF), and bone morphogenetic proteins (BMPs), play a key role in altering the microbiome by modulating tissue regeneration and immune responses [59]. The application of EMD and PDGF creates a regenerative environment by promoting the proliferation and differentiation of cells involved in tissue repair, such as fibroblasts and osteoblasts [60]. This improved healing environment reduces inflammation, which decreases the levels of pathogenic bacteria. Additionally, biologics like EMD mimic natural proteins involved in tooth development, providing a matrix for tissue regeneration [61]. This shift favors the growth of health-associated bacteria such as *Streptococcus mitis*, *Streptococcus gordonii*, and *Rothia dentocariosa*, which are essential for biofilm stabilization and tissue repair. These bacteria contribute to the formation of a healthier biofilm by producing enzymes and metabolites that inhibit the growth of pathogens and support tissue healing [62]. Antimicrobial agents, such as chlorhexidine and sub-antimicrobial dose doxycycline (SDD), reduce the bacterial load by directly targeting pathogenic species and disrupting biofilms [63]. Chlorhexidine and SDD work by disrupting bacterial cell membranes, inhibiting biofilm formation, and reducing virulence factor production [63]. Chlorhexidine disrupts bacterial cell walls and interferes with microbial adhesion, leading to cell death and biofilm disruption [64]. 

SDD inhibits matrix metalloproteinases (MMPs), which are enzymes involved in tissue breakdown and reduce the inflammatory response, indirectly decreasing the bacterial load [65]. 

After treatment, the reduction in pathogenic bacteria allows beneficial species such as *Streptococcus sanguinis* and *Actinomyces* to recolonize the area. These commensal bacteria contribute to a stable, health-associated biofilm by producing enzymes that inhibit pathogen colonization and promote tissue repair. The presence of these bacteria helps maintain a balanced microbiome and prevents the recurrence of periodontal disease [66]. Probiotics and prebiotics are increasingly being used in periodontal regeneration due to their ability to modulate the microbiome and support tissue healing. Probiotics, such as *Lactobacillus reuteri*, *Bifidobacterium*, and *Streptococcus salivarius*, are introduced to restore microbial balance by outcompeting pathogenic bacteria for adhesion sites and nutrients [67]. These beneficial bacteria produce lactic acid, which lowers the pH and inhibits the growth of pathogens. They also secrete bacteriocins and other antimicrobial peptides that directly kill pathogenic bacteria. In addition to their antimicrobial effects, probiotics can modulate the host immune response by reducing pro-inflammatory cytokines and enhancing the production of anti-inflammatory mediators, promoting tissue healing [68]. Prebiotics, which serve as nutrients for beneficial bacteria, further support the growth of these probiotics, helping restore a balanced and healthy microbiome. This shift away from pathogenic bacteria and towards a health-associated microbiome contributes to the long-term success of periodontal regeneration procedures [69].

## 12. Advanced Therapeutic Strategies in Periodontal Regeneration

Therapeutic strategies in periodontal regeneration aimed at disrupting pathogenic biofilms are evolving with the integration of advanced techniques and materials designed to not only eradicate harmful microorganisms but also restore microbial balance [70]. One of the most promising approaches involves the use of antimicrobial peptides (AMPs), which mimic the natural defense molecules produced by the host immune system. AMPs can selectively target and disrupt bacterial cell membranes, particularly those of pathogens within biofilms while sparing beneficial commensal bacteria [71]. This selective targeting makes them a valuable adjunct to mechanical debridement in periodontitis treatment [71].

Another cutting-edge method is the development of nano-biomaterials and antimicrobial nanocoatings. These materials, often based on nanoparticles such as silver, zinc oxide, or titanium dioxide, are capable of penetrating biofilms and delivering localized antimicrobial effects without the systemic side effects of traditional antibiotics. Nanoparticles can also be engineered to provide a controlled release of therapeutic agents, allowing for sustained biofilm disruption over time [72]. Photodynamic therapy (PDT), which uses light-activated photosensitizers to produce reactive oxygen species that kill bacteria, is another non-invasive approach gaining popularity for its ability to target biofilms without damaging host tissues [73].

Enzyme-based therapies are an emerging area in periodontal treatment, specifically targeting the biofilm matrix to enhance the efficacy of conventional treatments like scaling, root planing, and antimicrobial therapies [70]. Biofilms, which are complex communities of microorganisms encased in extracellular polymeric substances (EPS), provide a protective barrier for periodontal pathogens such as *Porphyromonas gingivalis*, *Tannerella forsythia*, and *Treponema denticola*, shielding them from host immune responses and antimicrobial agents. By breaking down the EPS matrix, enzyme-based therapies can significantly increase the susceptibility of bacteria to treatment [74]. 

Dispersin B, one of the most studied enzymes, targets the polysaccharides in the EPS matrix. Dispersin B is derived from *Aggregatibacter actinomycetemcomitans*, a periodontal pathogen, and functions by hydrolyzing the glycosidic bonds in poly-N-acetylglucosamine (PNAG), a key component of the biofilm matrix. By degrading this structural element, Dispersin B destabilizes the biofilm, allowing for easier penetration by antimicrobials and improved bacterial clearance by the host immune system [75]. Studies have shown that when used in combination with conventional scaling and root planing or with antimicrobial agents like chlorhexidine, Dispersin B enhances the removal of biofilms and reduces bacterial survival rates in periodontal pockets [76,77,78].

DNase is another enzyme that targets the extracellular DNA (eDNA) component of the biofilm matrix. eDNA is a critical structural element in biofilms, providing stability and contributing to the cohesion of bacterial communities. DNase breaks down this eDNA, weakening the biofilm structure and making the resident bacteria more vulnerable to antibiotics and immune defenses [79]. Recent research indicates that DNase treatment can significantly enhance the efficacy of antimicrobial therapies by disrupting the biofilm’s protective barrier. In periodontal treatments, DNase has been used to enhance biofilm disruption in combination with traditional mechanical debridement and antibiotic regimens, showing promising results in reducing biofilm persistence and improving clinical outcomes [80,81].

In addition to Dispersin B and DNase, other enzymes such as lysozymes, alginate lyase, and proteases are being investigated for their roles in biofilm degradation. Lysozymes target the peptidoglycan in bacterial cell walls, contributing to the lysis of bacterial cells within biofilms, while alginate lyase breaks down alginate, another key component of biofilms formed by bacteria such as *Pseudomonas* species. Proteases degrade proteinaceous components of the biofilm matrix, further destabilizing the biofilm structure [82,83]. The use of these enzymes in combination with conventional periodontal therapies represents a significant advancement in biofilm management. By targeting the structural components of biofilms, enzyme-based therapies disrupt the protective matrix, making bacteria more accessible to antimicrobials and immune cells, ultimately improving the efficacy of periodontal treatments [84]. 

The future of enzyme-based therapies lies in the development of more targeted and specific enzymes, as well as novel delivery systems such as nanoparticles and hydrogels, which can ensure sustained release and activity of these enzymes in the periodontal pocket. As research progresses, enzyme-based therapies are expected to become a key component of a comprehensive approach to managing biofilm-related periodontal diseases [85,86].

## 13. Antifouling and Bactericidal Materials for Oral Microbiome Stability in Periodontal Regeneration

During periodontal regeneration procedures, there is a risk of further imbalance in the oral microbiome, which could potentially lead to additional complications [2]. To address this issue, researchers have explored the use of antifouling or bactericidal materials that can help maintain the stability of the oral microbiome [87]. The formation of an early biofilm begins with the adsorption of proteins onto solid surfaces to create the salivary-acquired pellicle, which allows initial colonizers to adhere. This is followed by the subsequent adherence of other oral pathogens to the already immobilized bacteria, a process known as cohesion or coaggregation, leading to the maturation of the biofilm [88]. Biomaterials with antifouling properties, which include protein repulsion and bacteria anti-adhesion, can protect surfaces from the invasion of early biofilm. Additionally, these antifouling properties prevent the accumulation of dead pathogens and bio-foulants on oral surfaces or dental materials, thus maintaining other biofunctions [86]. Modern antifouling materials are typically polymeric agents, but certain biomolecules and special metals also exhibit antifouling characteristics [22]. The use of antifouling and bactericidal materials plays a crucial role in maintaining the stability of the oral microbiome during periodontal regeneration procedures, such as guided tissue regeneration (GTR) and bone grafting. These materials help prevent the formation of pathogenic biofilms and support a healthy microbial balance, which is essential for successful tissue regeneration and healing [89] (Table 2).

### 13.1. Antifouling Materials

1. **Polymeric Antifouling Agents:** These materials prevent the initial adhesion of proteins and bacteria to surfaces. Examples include polyethylene glycol (PEG) and zwitterionic polymers, which create a hydrophilic surface that resists protein adsorption and bacterial attachment [90,91].
2. **Biomolecules:** Certain biomolecules, such as antimicrobial peptides and enzymes, have antifouling properties. These molecules can disrupt bacterial communication and biofilm formation, maintaining a stable and healthy oral microbiome [92].
3. **Special Metals:** Metals like silver and copper possess inherent antifouling properties due to their antimicrobial activity. These metals can be incorporated into dental materials to prevent bacterial colonization and biofilm formation [93] Figure 5.

### 13.2. Bactericidal Materials

1. **Silver Nanoparticles:** Known for their broad-spectrum antibacterial activity, silver nanoparticles can be incorporated into dental materials to provide long-lasting bactericidal effects. They release silver ions, which can disrupt bacterial cell membranes and inhibit bacterial growth [94,95].
2. **Chlorhexidine:** This antiseptic agent is widely used in dental applications for its effective bactericidal properties. Chlorhexidine can be incorporated into barrier membranes or bone graft materials to reduce bacterial contamination and support healing [96].
3. **Quaternary Ammonium Compounds (QACs):** QACs are potent antimicrobial agents that can be integrated into dental materials. They disrupt bacterial cell walls and membranes, preventing bacterial growth and biofilm formation [97].
4. **Antimicrobial Peptides:** These peptides can be used in conjunction with biomaterials to provide targeted bactericidal effects. They work by disrupting bacterial membranes and interfering with essential cellular processes [98] Figure 5.

### 13.3. Application in Periodontal Regeneration

**Guided Tissue Regeneration (GTR):** In GTR, the use of antifouling and bactericidal materials can help create a conducive environment for the regeneration of periodontal tissues. For example, barrier membranes incorporating silver nanoparticles or chlorhexidine can prevent bacterial infiltration and biofilm formation, promoting the proliferation of beneficial cells and tissues [99,100].

**Bone Grafting:** During bone grafting procedures, the incorporation of bactericidal agents like silver nanoparticles into the graft material can help prevent infection and ensure a healthy healing environment. Additionally, the use of polymeric antifouling agents can prevent the initial adhesion of pathogenic bacteria, reducing the risk of biofilm formation on the graft surface [101].

The integration of antifouling and bactericidal materials in periodontal regeneration procedures is essential for maintaining the stability of the oral microbiome. These materials help prevent the colonization of pathogenic bacteria, reduce biofilm formation, and support the proliferation of beneficial microbial communities. By ensuring a balanced and healthy oral microbiome, these materials contribute to the success of periodontal regeneration and improve overall oral health outcomes [102].

## 14. Conclusions

Periodontal regeneration is a complex and multifaceted process that necessitates a deep understanding of the intricate interactions between host tissues and microbial communities. The shift from a healthy to a diseased state in the oral microbiome, characterized by dysbiosis and the proliferation of pathogenic species, underscores the need for targeted therapeutic strategies. Regenerative therapies, including guided tissue regeneration and bone grafting, hold promise for restoring lost periodontal structures. However, these interventions also present challenges in maintaining microbial balance and preventing infection.

Incorporating antifouling and bactericidal materials into regenerative procedures represents a significant advancement in periodontal therapy. These materials can effectively prevent biofilm formation and support the stability of the oral microbiome, thereby enhancing the outcomes of regenerative treatments. Understanding the dynamics of microbial transmission, the impact of various regenerative techniques on the microbiome, and the potential of antimicrobial strategies is crucial for optimizing periodontal therapy and achieving long-term oral health.

Future research should focus on the continuous refinement of regenerative materials and techniques to further improve clinical outcomes. Additionally, a deeper exploration into the molecular mechanisms underlying microbial shifts during periodontal regeneration will provide valuable insights for developing more effective and holistic treatment approaches. By addressing both the structural and microbiological aspects of periodontal diseases, we can pave the way for more successful and sustainable periodontal therapies.

## 15. Limitations

This review primarily relies on previously published data, limiting the introduction of novel findings. The heterogeneity in study designs and microbiome analysis techniques across the reviewed studies complicates direct comparisons, especially in understanding the effects of different grafting materials on microbial shifts. While we aimed to provide clinical insights, the review may lack clear recommendations for practice, particularly in terms of microbial management during periodontal regeneration.

The focus on guided tissue regeneration (GTR) and bone grafting limits the generalizability of our findings to other regenerative techniques. Additionally, the exclusion of non-English studies and reliance on certain databases may have introduced selection bias. Many referenced studies have short-term follow-up, which prevents a full understanding of the long-term effects of these procedures on the oral microbiome.

Further research, especially longitudinal studies with standardized methods, is necessary to better understand the clinical implications of these microbial dynamics in periodontal regeneration.

## Figures and Tables

**Table 1:** The changes in the microbiome during periodontitis, bone grafting, and guided tissue regeneration, along with the suggested treatments for each condition.

Condition	Changes in Microbiome	Ecosystem Changes	Suggested Treatment
**Periodontitis**	Shift to dysbiotic community with increased pathogenic species (e.g., *Porphyromonas gingivalis*, *Tannerella forsythia*, *Treponema denticola*)	Increased inflammation, tissue destruction, deeper periodontal pockets	Use of antimicrobial agents, probiotics, mechanical debridement, and host-modulation therapy
**Bone Graft**	Potential contamination and shifts in microbial balance; increased pathogenic bacteria if graft material not properly sterilized	Colonization by surrounding microbial communities; potential biofilm formation	Proper sterilization of graft materials; use of antimicrobial strategies, probiotics to manage microbial shifts
**Guided Tissue Regeneration**	Transient imbalance with initial increase in pathogenic bacteria; possible biofilm formation on membranes	Disruption of natural barriers; introduction of foreign materials; exposure of deeper tissues	Incorporation of antifouling and bactericidal materials in membranes; antimicrobial mouthwashes; good oral hygiene practices

**Table 2:** Outlines different types of antifouling and bactericidal materials, their mechanisms of action, and their applications in periodontal therapy.

Material Type	Example Material	Mechanism of Action	Application in Periodontal Therapy
**Antifouling Agents**	Polyethylene glycol (PEG)	Prevents bacterial adhesion by creating a hydrophilic layer, reducing biofilm formation.	Used in coatings for dental implants to prevent biofilm formation and enhance implant longevity.
**Antifouling Agents**	Zwitterionic Polymers	Forms a zwitterionic surface that repels proteins and bacteria, reducing fouling and biofilm growth.	Applied in regenerative membranes to reduce bacterial colonization and support tissue healing.
**Bactericidal Agents**	Silver Nanoparticles	Disrupts bacterial cell membranes, induces oxidative stress, and kills bacteria upon contact.	Incorporated into dental materials and membranes to provide antimicrobial effects and reduce infection risk.
**Bactericidal Agents**	Chlorhexidine	Disrupts bacterial cell walls and interferes with microbial adhesion, leading to cell death and biofilm disruption.	Used as a rinse or in gels for post-surgical care, reducing microbial load and preventing biofilm formation.
